# History of periodontal treatment and risk for intrauterine growth restriction (IUGR)

**DOI:** 10.1186/s12903-018-0623-2

**Published:** 2018-09-29

**Authors:** Cande V. Ananth, Howard F. Andrews, Panos N. Papapanou, Angela M. Ward, Emilie Bruzelius, Mary Lee Conicella, David A. Albert

**Affiliations:** 1 Department of Obstetrics and Gynecology, College of Physicians and Surgeons, Columbia University, New York, NY 10032 USA; 2 Department of Epidemiology, Joseph L. Mailman School of Public Health, Columbia University, New York, NY 10032 USA; 3 Department of Biostatistics, Joseph L. Mailman School of Public Health, Columbia University, New York, NY 10032 USA; 4 New York State Psychiatric Institute, New York, NY 10032 USA; 5 Division of Periodontics, Section of Oral, Diagnostic and Rehabilitation Sciences, College of Dental Medicine, Columbia University, New York, NY 10032 USA; 6 Section of Population Oral Health, College of Dental Medicine, Columbia University, New York, NY 10032 USA; 7 Aetna Inc., Pittsburgh, PA 15220 USA

**Keywords:** Periodontal treatment, Fetal inflammatory response, Intrauterine growth restriction

## Abstract

**Background:**

To explore the hypothesis that maternal periodontitis is associated with increased risk for Intrauterine Growth Restriction (IUGR), we examined the risk of IUGR in relation to periodontal treatment before, during and after pregnancy.

**Methods:**

We conducted a retrospective cohort analysis of insurance claims data from 2009 to 2012 for women who delivered a singleton live birth (*n* = 32,168). IUGR was examined as a function of type and timing of dental treatment, adjusting for potential confounders in logistic regression. Sensitivity analysis evaluated the potential effects of unmeasured confounding.

**Results:**

Women who received periodontal treatment after delivery, indicating the presence of untreated periodontal disease during pregnancy, had significantly higher odds of IUGR compared to women who received no periodontal treatment (adjusted OR 1.5, 95% CI 1.2, 1.8).

**Conclusions:**

Periodontal treatment provided in the immediate postpartum period, a proxy for periodontitis during gestation, was associated with increased risk of IUGR.

**Electronic supplementary material:**

The online version of this article (10.1186/s12903-018-0623-2) contains supplementary material, which is available to authorized users.

## Background

In industrialized countries, about four-fifths of low birthweight infants are born preterm and a fifth of these preterm births are due to intrauterine growth restriction (IUGR) [1]. In the absence of congenital malformations and/or chromosomal anomalies, IUGR entails two distinct processes: constitutional smallness, or pathological growth restriction [2]. The prevalence of IUGR varies substantially across populations, but prevalence rates range between 3 and 7%. The etiology of IUGR remains undetermined, but several risk factors for the condition have been identified. These include advanced maternal age, increased parity, smoking during pregnancy, low pre-pregnancy body mass index and low gestational weight gain (due to low energy intake), short maternal stature, poor maternal nutrition, maternal race/ethnicity, and low socioeconomic status being some of the important risk factors [3]. Maternal, placental and fetal infections are strongly implicated in the development of IUGR.

Periodontal diseases are associated with transient bacteremia that may facilitate dissemination of oral bacteria to the uterus, with subsequent infiltration of the amniotic fluid and the umbilical cord and invasion of the placenta. It is believed that hematogenous transport of bacteria and/or pro-inflammatory mediators from sites of periodontal infection into the placenta, fetal membranes, and amniotic cavity induces pathological processes that lead to adverse perinatal outcomes, including IUGR [4–7]. Uteroplacental infection and inflammation are thought to play key roles in the etiology of IUGR [8], with fetal inflammatory response syndrome being characterized as the important cause of IUGR [9]. Collectively, these infections account for up to 15% of IUGR cases [10].

Periodontal infections are associated with an increased risk for adverse pregnancy outcomes, including preterm delivery, and preeclampsia, but whether this increased risk also applies to IUGR has not been established. Since infections play an important role in IUGR, we hypothesized that: (i) maternal periodontitis is associated with an increased risk for IUGR; and (ii) treatment for periodontitis in the immediate postpartum period signifies presence of untreated periodontitis during pregnancy, and is associated with elevated risk of IUGR. Furthermore, given the increased risk of recurrence of IUGR and the temporal persistence of periodontal infections, we hypothesized that parity will be an effect modifier of the association between periodontal infection and risk of IUGR.

We tested these hypotheses by examining medical and dental insurance records in a large cohort of 32,168 women, comparing rates of IUGR among women undergoing periodontal care, other types of dental treatment, and those receiving no dental treatment, before, during and after pregnancy.

## Methods

### Study design and data sources

This retrospective cohort study examined insurance records of women concurrently enrolled in medical and dental insurance plans through Aetna Inc., a nationwide, private health insurer. Aetna’s data warehouse holds claim information on members for a four-year period. We therefore restricted the analysis to women that delivered a singleton live-birth between January 2010 and December 2011, and then included claims data for those birth events up to one year before gestation and up to one year after the birth event. We chose to restrict the study to singleton gestations, since the etiology and risk factors for IUGR vary between singleton and multiple births. The study cohort was restricted to women who had both medical and dental insurance, who were between 13 and 50 years of age at the time of delivery, and for whom zip code level data and other covariates were available. The analytic sample included 32,168 women.

Using data from 212,427 dental claim records for the period January 2009 through December 2012, procedures performed before, during and after pregnancy were identified and classified using the Code on Dental Procedures and Nomenclature Current Dental Terminology (CDT) 2009–10 edition for procedures occurring before 2011, and the CDT 2011/2012 edition for procedures from 2011 onwards. Maternal oral treatment type included periodontal treatment, prophylaxis, other dental treatment, or no oral treatment of any kind during the study period. Periodontal treatment was provided by general dentists and periodontists. Oral prophylaxis procedures can be provided by dentists or dental hygienists, however in the United States most oral prophylaxis procedures are provided by dental hygienists. Periodontal treatment included surgical and non-surgical codes (Additional file 1: Table S1). Because the insurance database did not include indicators of conception, the date of conception was estimated from the date of birth. For analytic purposes, each dental treatment category was considered in relation to the time period in which it occurred: pre-conception, during gestation, or after birth. The insurance dataset contained diagnosis codes and treatment codes for medical care, however it was limited to treatment codes for dental services. In the United States, dentists are only required to provide treatment codes for reimbursement via a dental claim. Given that periodontal disease is a chronic condition, periodontal treatment occurring after delivery was considered to indicate that periodontitis was present and untreated during pregnancy.

Using data extracted from 2,622,764 medical claims records, for the period January 2009 through December 2012, presence of IUGR was assumed if one or more International Classification of Diseases (ICD) 9th revision codes indicating slow fetal growth and fetal malnutrition (764.x), or poor fetal growth affecting management of mother (656.5x) had been used. Fetal growth restriction was defined as an IUGR diagnosis within 14 days of delivery; thus, in this study, the term IUGR refers to growth restriction defined accordingly. In each record, both primary and secondary claims were searched. Multiple births were identified and eliminated from the analytic file using supplementary delivery codes V27.2 to V27.9.

Maternal income, race and ethnicity were not available in the insurance database. We therefore imputed these characteristics based on the zip code of residence for each woman at the time of insurance enrollment, and the 2010 United States Census zip code level data for these variables. We identified complications of pregnancy using ICD-9 codes (Additional file 1: Table S2). Because not all women had dental coverage for the entire study period, months of enrollment for each woman was included as a covariate. The sample included women from all 50 states, the District of Columbia, and Puerto Rico.

### Statistical analysis

The risk of IUGR was associated with types of dental and periodontal treatment, as previously defined. We fit logistic regression models from which we derived odds ratios (OR) and 95% confidence intervals (CI) to assess the magnitude of the effect. In these models, we adjusted for potential confounding factors including pregnancy complications, maternal age at delivery, primiparity, zip code level income, race, and ethnicity; the models were also adjusted for duration of continuous dental coverage during the study period. Annual income was analyzed in quintiles, and categorized in US dollars as ≤28,125; 28,126 to 33,500; 33,501 to 39,283, 39,284 to 48,831; and ≥ 48,832. To assess non-linear effects of maternal age on the odds of incident IUGR, we included a quadratic term for age in the regression models.

To test for parity-related effect modification, a two-way interaction term between periodontal treatment and primiparity was included in the logistic regression model. Since the Wald-type chi-square test for the interaction term was significant (*P* < 0.01), we present the results of models stratified by parity.

To determine whether risk of IUGR increases with severity of periodontitis, we examined the rate of IUGR in relation to the number and type of periodontal treatment procedures, and compared women who received surgical as opposed to non-surgical periodontal treatment.

### Sensitivity analysis

Since the association between periodontal treatment and IUGR is likely affected by unmeasured confounding, we undertook a sensitivity analysis to evaluate the extent to which unmeasured confounding may have impacted the associations [11]. This sensitivity analysis was based on the following assumptions: (i) the prevalence rates of the unmeasured confounder among pregnancies with and without IUGR were 3% and 6%, respectively; and (ii) the odds ratio of IUGR comparing the presence versus absence of unmeasured confounding was allowed to vary between 0.1 (protective) to 1.0 in 0.1 increments, and 2.0 (increased risk) to 10 in 1.0 increments. The evaluation of the impact of unmeasured confounding was based on fairly conservative assumptions.

## Results

Of the 32,168 women in the study, 18,593 (57.8%) received some form of dental treatment during the study period; 9.0% (*n* = 2895) received periodontal treatment, 41.2% (*n* = 13,246) received prophylaxis and 7.6% (*n* = 2452) received some other form of dental treatment (Table 1). The mean maternal age at the time of delivery was 30.8 (standard deviation (SD) = 5.6); subjects lived in zip codes with median Black population of 5% (range 0–99), and median Hispanic population of 8% (range 0–100). Complications of pregnancy were documented for 33.9% of the women in the study. On average, women were enrolled in a dental plan for 27.9 months (SD = 13.3) during the study period. There were relatively small differences between dental treatment groups with respect to each of the above covariates.

**Table 1:** Distribution of maternal sociodemographic characteristics by dental treatment type (*n* = 32,168)

Maternal characteristics	Total (%)	No dental treatment (%)	Periodontal treatment (%)	Prophylaxis (%)	Other dental treatment (%)	*P*-value
Number of subjects (%)	32,168 (100.0)	13,575 (42.2)	2895 (9.0)	13,246 (41.2)	2452 (7.6)	
Maternal age (years)						< 0.01
< 20	2.6	3.4	1.6	1.9	2.7	
20–24	10.8	12.5	13.9	7.6	15.3	
25–29	26.0	26.9	31.8	23.1	29.9	
30–34	34.3	32.7	28.5	37.7	31.5	
≥ 35	26.3	24.4	24.2	29.6	20.6	
Mean age (SD)	30.8 (5.6)	30.4 (5.8)	30.2 (5.5)	31.6 (5.3)	29.9 (5.7)	< 0.01
Primiparity	32.2	31.4	31.8	33.5	30.0	< 0.01
Household Annual income (US $; quintile)						< 0.01
1st (≤28,125)	6.3	6.7	9.0	5.2	6.6	
2nd (28,126 to 33,500)	7.3	7.8	10.5	5.8	8.4	
3rd (33,501 to 39,283)	10.5	11.0	10.7	9.5	13.4	
4th (39,284 to 48,831)	20.6	20.3	22.7	19.9	23.4	
5th (≥48,832)	55.3	54.2	47.2	59.6	48.1	
Median income (US $; range)	50,991 (5000 to 200,000)	50,555 (5787 to 209,001)	47,153 (7436 to 200,001)	52,800 (5000 to 173,368)	47,683 (7236 to 185,466)	< 0.01
Black race (%)						< 0.01
≤ 10	70.0	66.3	71.7	73.8	67.9	
11–35	20.5	21.8	19.7	19.0	22.8	
36–50	2.8	3.1	2.9	2.4	3.6	
≥ 51	6.6	8.8	5.6	4.7	5.8	
Median (range) %	5 (0, 99)	6 (0, 99)	5 (0, 98)	5 (0, 99)	5 (0, 98)	< 0.01
Hispanic ethnicity (%)						< 0.01
≤ 5	40.4	39.1	31.8	44.4	36.8	
6–10	19.8	18.9	22.8	20.1	19.5	
11–20	18.3	18.6	18.9	17.9	18.5	
21–42	13.0	13.3	16.1	11.6	15.0	
≥ 43	8.4	10.1	10.3	6.0	10.2	
Median (range) %	8 (0, 100)	8 (0, 100)	9 (0, 99)	7 (0, 100)	9 (0, 97)	< 0.01
Complications of pregnancy (%)^a^	33.9	34.0	34.6	33.1	36.5	< 0.01
Dental enrollment months, Median (range)	28 (1, 46)	22 (1, 46)	33 (1, 46)	32 (1, 46)	29 (1, 46)	< 0.01

^a^Details of the pregnancy complications are shown in Additional file 1: Table S2

Table 2 shows the frequency of each type of periodontal treatment in the cohort. The most frequent non-surgical periodontal procedure was scaling and root planing, which was documented for 7.4% (*n* = 2388) of the women. Full mouth debridement (*n* = 584, 1.8%) and localized delivery of chemotherapeutic agents (*n* = 229, 0.7%) were less frequent. Surgical periodontal procedures occurred only rarely. For over two thirds of the 2895 women who received periodontal treatment during the study period, that treatment occurred only in the period after birth (*n* = 1956, 67.6%). A total of 440 (15.2%) received dental treatment only during gestation, and 343 (11.8%) received treatment in the period prior to conception. 753 (2.6%) received treatment in the gestation and post-gestation periods, 550 (1.9%) received treatment in the pre-gestation and post-gestation periods, and 232 (0.8%) received treatment in the pre-gestation and gestation periods.

**Table 2:** Frequency (%) of periodontal surgical and non-surgical procedures among women in sample (n = 32,168)^a^

Procedure category	Number	% of total sample
Surgical procedures		
Gingival flap	11	< 0.1
Osseous surgery	40	0.1
Bone replacement graft	58	0.2
Tissue regeneration procedure	61	0.2
Non-surgical procedures		
Scaling and root planing	2388	7.4
Full mouth debridement	584	1.8
Localized chemotherapeutic agents	229	0.7

^a^Number of women who had at least one instance of each procedure; a woman may have had more than one procedure but is counted once in each procedure category

IUGR was documented in 2027 fetuses (6.3%). The association between dental treatment before, during and after gestation and the risk of IUGR is shown in Table 3. The incidence of IUGR was 9.2% (*n* = 192) among those receiving periodontal treatment after delivery and 6.1% (*n* = 1835) for those receiving no periodontal treatment. The odds of IUGR for those receiving periodontal treatment post-gestation compared to those receiving no periodontal treatment was 1.5 (95% CI 1.2, 1.8) following adjustment for confounders. The odds of IUGR was elevated among multiparous women who received periodontal treatment post-gestation (OR 1.6, 95% CI 1.3, 1.9). Among primiparous women who received periodontal treatment post-gestation, the risk of IUGR was not elevated (OR 1.3, 95% CI 1.0, 1.8).

**Table 3:** Risk of intrauterine growth restriction (IUGR) in relation to timing of dental treatment (n = 32,168)

Treatment	Period of treatment	Number with claims	IUGR n (%)	Adjusted odds ratio (95% confidence interval)		
				Overall	Primiparous women	Multiparous women
Periodontal	Pre-gestation	428	22 (5.1%)	0.7 (0.4, 1.1)	0.5 (0.2, 1.1)	0.9 (0.5, 1.5)
	Gestation	540	40 (7.4%)	1.2 (0.8, 1.7)	1.4 (0.8, 2.4)	1.1 (0.7, 1.7)
	Post-birth	2088	192 (9.2%)	1.5 (1.2, 1.8)	1.3 (1.0, 1.8)	1.6 (1.3, 1.9)
Prophylaxis	Pre-gestation	3387	220 (6.5%)	1.0 (0.8, 1.3)	1.0 (0.7, 1.6)	1.0 (0.7, 1.4)
	Gestation	7043	409 (5.8%)	1.0 (0.8, 1.2)	1.1 (0.8, 1.6)	0.9 (0.7, 1.2)
	Post-birth	11,743	743 (6.3%)	1.0 (0.8, 1.2)	0.9 (0.7, 1.2)	1.0 (0.9, 1.3)
Any dental	Pre-gestation	4655	306 (6.6%)	1.2 (0.9, 1.5)	1.0 (0.7, 1.5)	1.2 (0.9, 1.7)
	Gestation	8986	535 (6.0%)	0.9 (0.7, 1.1)	0.8 (0.6, 1.2)	0.9 (0.7, 1.2)
	Post-birth	14,969	986 (6.6%)	1.1 (0.9, 1.3)	1.2 (0.9, 1.6)	1.1 (0.9, 1.3)

Odds ratios were adjusted for the confounding effects of maternal age, maternal age-square, household income, proportions of African-American and Hispanic ethnicities, and complications of pregnancy. An individual subject may have had a claim in more than one time period, and/or may have received more than one type of treatment in a given time period; the regression models adjust for these instances of multiple treatment exposures

The rate of IUGR among those with no dental treatment at any time before, during or after pregnancy was 6.0%, which was marginally but significantly lower than the IUGR rate of those who received any treatment (6.5%; *p* = 0.048); there was also a marginally significant difference in IUGR rates in the post-gestational period between those who received no dental treatment in this period (6.1%) and those who received some form of dental treatment in this period (6.6%; *p* = .049). The slightly elevated IUGR rates of those receiving any form of dental treatment is clearly driven by the increased IUGR rates associated with periodontal treatment, as shown in Table 3.

### Sensitivity analysis for unmeasured confounding

**Fig. 1:**
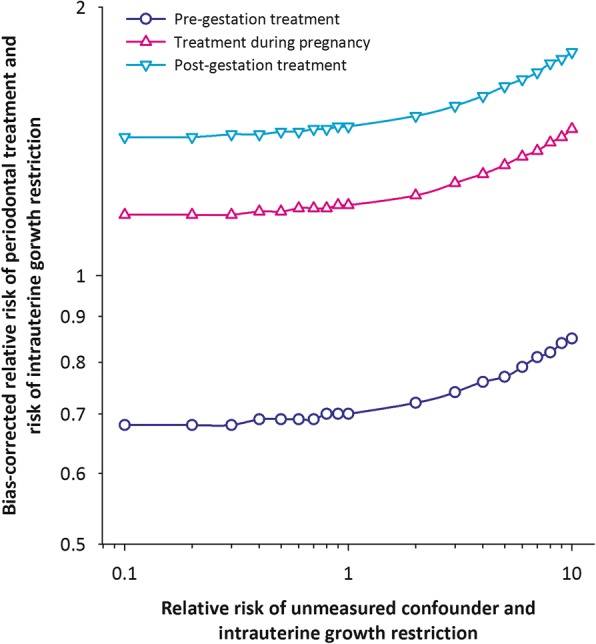
Sensitivity Analysis for Unmeasured Confounding Between Periodontal Treatment Before, During and Post-Gestation and IUGR. Sensitivity analysis to evaluate the impact of unmeasured confounding of the association between periodontal treatment before (top panel), during (middle panel), and post-gestation (bottom panel) and IUGR. The observed confounder-adjusted odds ratio and 95% confidence interval are also shown for each panel. The unmeasured confounding bias-corrected odds ratio of IUGR for each of the three periodontal treatment periods are shown for prevalence estimates varying from 0.5 to 6.0% of the unmeasured confounder among both the IUGR and non-IUGR groups. The odds ratio of IUGR in relation to the unmeasured confounder is assumed to be 1.25. The red circle for each panel shows the bias-corrected odds ratio for one scenario of the prevalence of the unmeasured confounder of 2% and 4% among IUGR and non-IUGR groups, respectively, and the odds ratio of IUGR in relation to the unmeasured confounder of 1.25. The bias-corrected odds ratio for each of the three scenarios are 0.9 (95% CI 0.6, 1.5) for pre-gestation periodontal treatment, 1.6 (95% CI 1.1, 2.3) for periodontal treatment during pregnancy, and 2.0 (95% CI 1.6, 2.3) for periodontal treatment post-gestation

The odds ratios corrected for unmeasured confounder(s) are shown in Fig. 1. For instance, if the odds ratio of IUGR comparing the presence versus absence of an unmeasured confounder was 2.0, the bias-corrected odds ratio for each of the three scenarios were 0.7 (95% CI 0.5, 1.1) for pre-gestation periodontal treatment, 1.2 (95% CI 0.9, 1.8) for periodontal treatment during pregnancy, and 1.5 (95% CI 1.3, 1.8) for periodontal treatment post-gestation. For odds ratios of the unmeasured confounder over 5, the bias-corrected odds ratio were enhanced for both periodontal treatment during pregnancy and post-gestation. These findings confirm the confounder-adjusted odds ratios reported earlier (Table 3), and when unmeasured confounding is taken into account, the associations between periodontal treatment both during pregnancy and post-gestation are associated with increased odds of IUGR.

To determine whether severity of post-gestation periodontal care was associated with the likelihood of IUGR, we developed a four-category measure capturing the number of claims for post-gestational maternal periodontal care: zero, one, two, and three or more. The rate of IUGR increased from 6.1% for those with no periodontal care to 8.1% among those with 1 instance of periodontal treatment, 9.8% for those with 2 instances of periodontal treatment, and peaking at 11.1% for those with 3 or more instances of periodontal treatment after birth (*P* < 0.01).

## Discussion

The results indicate an association between maternal periodontal disease and odds of IUGR. We observed significantly elevated odds of IUGR among women who experienced periodontal disease during pregnancy, as evidenced by periodontal treatment shortly after giving birth. We also found that delivery of a higher volume of periodontal treatment, a possible indicator of more severe periodontal disease, was associated with increased incidence of IUGR. The effect was strongest among those who received periodontal treatment after giving birth, an indication of untreated periodontal disease during pregnancy. While relatively few women received periodontal treatment during pregnancy, the sensitivity analysis suggested that there may be elevated risk of IUGR during this period as well. However, contrary to our initial hypothesis, the risk of IUGR in relation to the timing of the receipt of dental treatment did not vary by parity.

### Limitations of the data

Diagnosis of IUGR was based on ICD coding, and this may have introduced some misclassification. Women carrying IUGR fetuses, particularly those that are diagnosed as not being severely growth restricted, are less likely to undergo clinician-initiated obstetrical intervention (labor induction or a prelabor cesarean) and less likely to have a diagnosis of IUGR recorded [12]. However, for severe IUGR (e.g., estimated fetal weight below the third or the first percentiles), misclassification of IUGR status is very unlikely since growth restriction serves as a sentinel cause for obstetrical intervention, and is therefore billed for insurance reimbursement. For the same reason, we believe the recording of the exposure is accurate in this data system because periodontal treatment is the basis for a reimbursable claim.

Second, despite adjustment for several confounders, we lack data on smoking and maternal pre-pregnancy body-mass index. However, the sensitivity analysis conducted to determine the potential effect of unmeasured confounding indicates that our models are robust. Finally, dental data is based on CDT codes that reflect treatment of periodontal disease rather than diagnosis. However, we believe that the treatment codes are sufficiently specific to infer presence of periodontal disease. While women included in this study are from virtually all states in the US, the insured populations are from middle to higher-income socioeconomic strata. This should be considered while generalizing the results from the study; however, it is unlikely that the association between IUGR and infections in general, and periodontal disease in particular, would be any lower among poorer women than among those we studied.

It may appear anomalous in terms of causal reasoning that the relationship we report is between an outcome (IUGR) that occurs *prior* to an exposure (periodontal treatment in the period immediately after birth). However, periodontal disease is a chronic condition. Therefore, it is plausible to assume that women who were treated in the immediate post-gestation period experienced periodontitis and its systemic impact during gestation.

Finally, while we report that there is an increase in the rate of IUGR as a function of increasing number of treatments for periodontal disease, there are many factors that determine frequency of treatment; therefore, this finding is only suggestive of a relationship between the severity of periodontal disease and increased risk of IUGR.

### Strengths of the study

In this study, a large sample of integrated medical and dental claims data provided a unique opportunity to explore the association between IUGR and periodontitis. In addition, the findings appear robust following adjustment for observed confounders, in fact correction for unmeasured confounding makes the associations stronger. Conducting secondary analyses using insurance data to shed light on the possible causes of negative birth outcomes is highly economical, and valuable in suggesting directions for future research.

### Biological interpretations

The finding that periodontal treatment post-gestation was associated with an increased risk of IUGR in a large national sample add to the growing body of literature indicating a relationship between periodontal infection and related inflammation with adverse birth outcomes [4–8, 13]. Periodontal treatment in the period immediately following gestation is interpreted as signifying that periodontal disease was present during gestation. The inflammatory process associated with periodontal disease and the presence of periodontal pathogens in the blood can affect the fetus and the placenta [4].

This study utilized periodontal treatment as a proxy for the presence of periodontal disease. Periodontal treatment during gestation was expectedly rare and was also low during the pre-gestational period; our sample size was therefore too small to evaluate effects of treatment during pre-gestation and gestation. We expect that treatment during the pre-gestational and gestational periods to have limited adverse impact on birth outcomes. Tonetti and colleagues observed a short term increase in the systemic inflammatory response immediately following periodontal treatment which was then followed by a decrease in inflammation [14]. The finding that deleterious effects associated with periodontal therapy are short-lived is consistent with our finding of no statistically significant effect of treatment in the period prior to gestation and during gestation. However, periodontal treatment provided immediately following birth, which we found to be significantly related to IUGR appears to be a marker of disease during gestation.

Boggess and colleagues also observed that the incidence of small for gestational age increased with periodontal disease severity [15]. These findings are consistent with observations by several other investigators. In a study of Brazilian women, Siqueria and colleagues found increased odds of IUGR (adjusted OR 2.06, 95% CI 1.07, 4.19) among women diagnosed with periodontitis [16]. Similarly, Kumar and colleagues reported an increased association between periodontitis and IUGR, which was attenuated after adjusting for confounders [17]. The associations that we report are very similar to those of the Brazilian study.

The insured and employed population in our analyses is in the upper quartile of income in the United States and would be expected to have better oral hygiene and prevention practices. In addition, it is expected that utilizing treatment as a proxy for periodontal disease to some extent underestimates the true incidence of periodontal disease. In our analytical sample, maternal periodontal disease, indirectly assessed through the delivery of periodontal treatment in the immediate post-partum period, affected 9% of the women. By comparison, earlier studies have reported prevalence rates of 56–61% for maternal periodontitis [18, 19]. It should be noted that while we and Siquiera and colleagues [16] observed an increased odds for growth restriction with periodontal disease, other studies did not find such a relationship [20].

## Conclusions

Periodontal disease manifests itself as destruction of the supporting structures of the teeth and is associated with systemic dissemination of bacteria and bacterial products as well as the release of inflammatory mediators that can adversely impact the placenta resulting in fetal growth restriction. In our analysis, IUGR was present in 6.3% of the sample. In 2012, 46% of the United States adult population was estimated to have experienced periodontal disease [21]. The high prevalence of periodontal disease in adults, and the cost associated with the morbidity and mortality of adverse birth outcomes, justifies further investigation of the systemic impact of periodontal infection/inflammation on the feto-placental unit.

As demonstrated in this study, research that involves the integration of medical and dental records can be informative in elucidating the role of potential exposures on adverse outcomes, and may ultimately lead to improved patient outcomes and more cost-effective care [22]. In particular, the use of combined medical and dental national insurance claims data provides an opportunity to explore the association of birth outcomes with dental health and dental treatment in women of childbearing age. 56% of American adults aged 19–64 had private dental insurance in 2009, and 10% of all procedure types in the dental office were related to periodontics [23, 24]. Data were obtained from a national insurance carrier that provides medical insurance to 23.5 million persons, and dental coverage to about 14.6 million persons across the United States [25].

In this retrospective study we show an association between periodontal treatment as a proxy for the presence of periodontal disease and IUGR, however randomized controlled trials are needed to establish the efficacy of periodontal therapy on pregnancy outcomes such as fetal growth restriction. Periodontal care should be emphasized for women of childbearing age to improve general oral health. Policies encouraging evaluation and early intervention to control/eliminate periodontal pathology prior to pregnancy may be able to reduce the risk of IUGR and related complications in the newborn.

## Additional file

Additional file 1: **Table S1.** Current Dental Terminology CDT Codes. **Table S2.** Pregnancy Complications and Disease Comorbidities. (DOCX 36 kb)

## Abbreviations

CDT: Code on Dental Procedures and Nomenclature Current Dental Terminology
CI: Confidence Intervals
ICD: International Classification of Diseases
IUGR: Intrauterine Growth Restriction
OR: Odds Ratio
SD: Standard Deviation
