# Supplementary material for: History of periodontal treatment and risk for intrauterine growth restriction (IUGR)
Source: BMC Oral Health. 2018 Sep 29;18:161. doi: 10.1186/s12903-018-0623-2 (PMC6162907; doi:10.1186/s12903-018-0623-2)
Supplement: Supplementary file 1 — Table S1. Current Dental Terminology CDT Codes. Table S2. Pregnancy Complications and Disease Comorbidities. (DOCX 36 kb) [file 12903_2018_623_MOESM1_ESM.docx]

**Table S1**

**Current Dental Terminology CDT Codes**

| **Dental treatment category** | **CDT-3 Codes** |
| --- | --- |
|  |  |
| Periodontal treatment - Surgical | 4220, 4245, 4260, 4263, 4264, 4266, 4267, 4268, 4274 |
| Periodontal treatment – Non-surgical | 4341, 4342, 4355, 4381 |
| Oral prophylaxis | 1110 |
|  |  |
| **Dental Treatment Category** | **CDT-4 Codes** |
| Periodontal treatment - Surgical | 4240, 4241, 4245, 4260, 4261, 4263, 4264, 4265, 4266, 4267, 4268, 4274 |
| Periodontal treatment – Non-surgical | 4341, 4342, 4355, 4381 |
| Oral prophylaxis | 1110 |
|  |  |

**Table S2**

**Pregnancy Complications and Disease Comorbidities**

| **Category** | **ICD-9 codes** |
| --- | --- |
|  |  |
| **Complications related to pregnancy** |  |
| Renal Disease | 646.20-646.23 |
| Habitual aborter | 646.3, 646.30-646.33 |
| Liver Disorders | 646.7, 646.70-646.73 |
| Thyroid Dysfunction | 648.1, 648.10-648.13 |
| Anemia | 648.2, 648.20-648.23 |
| Drug Dependence | 648.3, 648.30-648.33 |
| Mental Disorders | 648.4, 648.40-648.43 |
| Congenital Cardiovascular Disorders | 648.5, 648.50-648.53 |
| Other Cardiovascular Diseases | 648.6, 648.60-648.63 |
| Tobacco Use Disorder | 649.00-649.04 |
| Obesity | 649.10-649.13 |
| Spotting | 649.50-649.53 |
| Isoimmunization | 656.1, 656.10-656.23 |
| Abnormality in Fetal Heart Rate | 659.7, 659.70-659.73 |
| Pyrexia of Unknown Origin During Puerperium | 672, 672.0, 672.00 |
| Obstetrical Pyemic | 673.3, 673.30-673.33 |
| Other Pulmonary Embolism | 673.8, 673.80-673.83 |
| Cerebrovascular Disorders in Puerperium | 674.0, 674.00-674.04 |
| Peripartum Cardiomyopathy | 674.50-674.53 |
| Pregnancy with History of Pre-term Labor | V23.41 |
| Pregnancy with Poor Reproductive History | V23.5 |
| Insufficient Prenatal Care | V23.7 |
| Supervision of Other High-risk Pregnancy | V23.8 |
| **Disease Comorbidities** |  |
| Diabetes Mellitus | 250.0, 250.00-250.93, 357.2, 362.0, 362.01-362.07, 366.41, 648.0, 648.00-648.03, 648.8, 648.80-648.83, V45.85, V53.91, V58.67, V55.46 |
| Hypertension | 401, 401.0, 401.1, 401.9, 402.0, 402.00, 402.01, 402.1, 402.10, 402.11, 405, 405.0, 405.09, 405.1, 405.19, 405.9, 405.99, 642, 642.0, 642.00-642.73, 642.9, 642.90-642.93 |
| Systemic Lupus Erythematosus | 710.0 |
| Endocrine Disorder | 278, 278.01 |
|  |  |
